# Quality of medical services provided to mothers, newborns and children at the hospital level in the Kyrgyz Republic

**DOI:** 10.7189/jogh.16.04109

**Published:** 2026-03-20

**Authors:** Indira Zholdosheva, Gulmira Nazhimidinova, Begaiym Akmatova, Rabiia Allakhveranova, Nurshaim Tilenbaeva, Martin W Weber, Sophie Jullien

**Affiliations:** 1 International Higher School of Medicine, Bishkek, Kyrgyz Republic; 2 Kyrgyz State Medical Institute for Continuing Education, Bishkek, Kyrgyz Republic; 3 Central Asian International Consulting, Research and Development Department, Bishkek, Kyrgyz Republic; 4 World Health Organization Athens Office on Quality of care and patient safety, World Health Organization Regional Office for Europe, Athens, Greece

## Abstract

**Background:**

The Kyrgyz Republic has recently implemented health programmes to improve the quality of care for mothers, newborns and children. To support these efforts, a three-year World Health Organization (WHO) quality improvement (QI) project aimed to strengthen clinical practices and service delivery. This study was conducted to independently assess the project’s effectiveness and inform policy and programming.

**Methods:**

Data were collected retrospectively from 18 hospitals: nine that implemented the intervention (IH) and nine control hospitals (CH). Medical records were randomly selected for women in labour, newborns and children hospitalised in 2019, 2021 (pre-QI project), and 2023 (post-QI project).

**Results:**

We reviewed 1707 women’s, 1736 newborns’, and 1593 children’s records. The proportion of women with a planned caesarean section before 39 + 0 weeks of gestation was 44.8% (2021) and 28.3% (2023) in IH, and 53.3% and 50.0%, respectively, in CH. Antibiotic prophylaxis use for caesarean sections was high in both IH and CH. The proportion of newborns breastfed within the first 30 minutes of life in IH was 58.3% (2021), and 50.6% (2023), and in CH 56.6% and 64.1%, respectively. Newborns were unnecessarily prescribed antibiotics in IH (13.7% in 2021, 16.2% in 2023), and in CH (24.2% and 6.1%, respectively). Children were frequently prescribed unnecessary antibiotics both in IH and CH. Children with pneumonia were unnecessarily prescribed corticosteroids both in IH (35.5% in 2019, 54.7% in 2023) and in CH (28.3% in 2019, 50.9% in 2023). The proportion of children with diarrhoea receiving oral rehydration salts (ORS) and zinc increased between the start and the end of the QI project in IH while this was not the case for zinc prescription in CH.

**Conclusions:**

These results highlight the importance of continuous monitoring and targeted interventions to enhance quality care. Routine clinical audits based on medical record reviews should be institutionalised to support hospital managers in enhancing clinical practices.

Over the past 15 years, there has been a steady decline in infant mortality and child mortality under five years of age in the Kyrgyz Republic [1]. The Kyrgyz Republic is among the countries that have achieved the Millennium Development Goals (MDG 4) to reduce infant and child mortality by two thirds compared to 1990 [2]. Maternal mortality in 2020 was 42.4%, by the end of 2023 it decreased to 26.0%. Child mortality in 2019 was 17.4, by the end of 2023 it decreased to 16.6. The neonatal mortality rate in was 12.2 in 2021 and 11.5 in 2023 [3].

The Kyrgyz Republic is a lower middle income landlocked country in Central Asia. The population is 7 037 000 people, of which 3 557 000 women, including up to 1 800 000 women of reproductive age, and 790 000 children under five years of age [3]. The birth rate is 24.0 per 1000 population in 2020, by 2023 it was decreased to 21.5 [4].

The WHO assessment of sexual, reproductive, maternal, newborn, child and adolescent health in the context of universal health coverage in Kyrgyzstan recommends: ‘evidence-based guidelines and protocols on when to admit and refer patients to hospital should be followed and steps should be taken to implement them at both the Primary Health Care and hospital levels’ [5]. As part of this recommendation for standardising service delivery, the ‘Pocket Book of Hospital Care for Children’ [6] has enabled early detection of illnesses in children, prompt referral for hospitalisation and more rational use of antibiotics. The country has also implemented World Health Organization (WHO) standards for the care of women during pregnancy, childbirth and the postpartum period (Integrated Management of Pregnancy and Childbirth (IMPAC)), including early detection of complications and timely referral for inpatient treatment [7].

In 2021–2023, a WHO project on ‘Improving the quality of hospital care to reduce maternal, newborn and child deaths and accelerate the achievements of the Sustainable Development Goals (SDGs) health targets’ took place in four countries including the Kyrgyz Republic. A series of multi-level interventions were implemented in nine hospitals across the country, including training, supportive supervision, strengthening quality improvement committees, and semi-annual collaborative quality improvement meetings between hospitals. The aim of this research was to assess the effectiveness of the multi-level interventions of the WHO project for improving the quality of hospital care provided to mothers, newborns and children.

## METHODS

### Study design

We employed an interrupted time series design with a control arm. We collected data in the nine intervention hospitals where the WHO quality improvement (QI) project was implemented. As controls, we selected nine hospitals not included in the WHO project, matched by location and level of care as a comparator. More information on the QI project is detailed elsewhere [8].

We retrospectively sampled and reviewed medical records to collect data on indicators of care at three time points: 2019, before the intervention and before the COVID-19 pandemic, 2021 corresponding to the beginning of the QI project, but towards the end of the COVID-19 pandemic, which might have affected care, and 2023 at the end of the QI project. For each time point, we sampled 40 records from August, September and October for each of the groups: women hospitalised for childbirth, newborns after birth, and hospitalised children. We chose the number of 40 medical records based on similar work conducted in the Region [9,10]. The data collection period was selected to obtain the most recent data from each period, coinciding with the start of the QI project in October 2021 [8,11].

### Inclusion criteria

For maternal and neonatal care, all women hospitalised for term delivery (from 37 + 0 weeks) and all newborns born at the hospital staying in the physiological department were eligible. For paediatric care, we included children aged two to 59 months hospitalised with a primary diagnosis of acute respiratory infection, pneumonia, acute bronchitis/bronchiolitis, or acute gastroenteritis.

### Primary outcomes

Primary outcomes included, for women, indication of caesarean section, unjustified use of antibiotics, and uterotonic administration within one minute after delivery; for newborns breastfeeding within 30 minutes after birth, and unjustified use of antibiotics; and for children, unjustified use of antibiotics and corticosteroids, prescription of oral rehydration salts (ORS) and zinc, and use of pulse oximetry (Table S1 in the **Online Supplementary Document**). We selected these validated indicators from the WHO standards for improving quality of care for mothers, newborns [12], children and young adolescents [13], based on their relevance for the countries and on the key findings reported in the previous QI project in the country [9,10]. The WHO pocket book of hospital care for children provides standards of care for common and severe diseases in childhood [6].

### Selection of medical records

Every third medical record was systematically selected from the stack until 40 records were available for data collection. Medical records corresponded to hospitalisations of women, newborns, and children during August, September, and October of each data collection year. In hospitals where medical records were kept separately for women who gave birth vaginally and women who gave birth by caesarean section, 20 medical records were selected for each of the two groups. Similarly, in hospitals where medical records were kept separately for children with acute respiratory infections and children with diarrhoea who were admitted to different wards, 20 medical records were selected for each of the two groups.

### Data collection and analysis

A research team including obstetricians-gynaecologists, neonatologists and paediatricians collected data on the following provision of care indicators. For women we collected data on caesarean section, use of antibiotics, and uterotonic administration within one minute after delivery. For newborns we collected data on breastfeeding within 30 minutes after birth, and unjustified use of antibiotics. For children, we collected data on prescription of antibiotics, corticosteroids, oral rehydration salts (ORS) and zinc, and use of pulse oximetry. We selected these indicators based on the key findings reported in the previous QI project in the country and the WHO global standards [9,10,12,13].

Data were directly inserted in excel tables specifically prepared for study data collection.

For each of the groups (mothers, newborns, children) we calculated proportions for each indicator, and we looked at trends over the years looking both at intervention and control hospitals, using Stata V.16.0 (College Station, Texas, USA).

## RESULTS

For women, we reviewed a total of 1707 medical records, including 827 from intervention hospitals and 880 from control hospitals. For newborns, we included 1736 medical records (846 from intervention hospitals, and 890 control hospitals). For children, we reviewed 1593 medical records, including 837 from intervention hospitals and 756 from control hospitals (Figure S1 in the **Online Supplementary Document**).

### Maternal care

**Figure 1:**
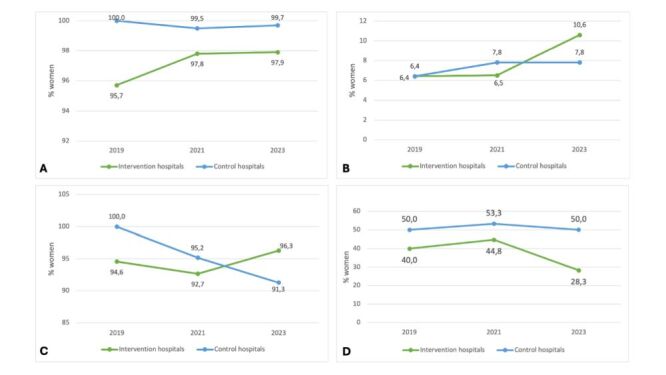
Quality indicators for maternal care. **Panel A.** Uterotonics in the first minute after vaginal delivery. **Panel B.** Use of antibiotics in vaginal delivery. **Panel C.** Prophylactic antibiotics in caesarean section. **Panel D.** Planned caesarean section before 39 + 0 weeks of gestation.

In 2019, the use of uterotonics within one minute of delivery was 100% in the control hospitals and 95.7% (n/N = 111/116) in the intervention hospitals. In 2021 and 2023, the proportion of women receiving an uterotonic within one minute of delivery was 97.8% (n/N = 349/353) and 97.9% (n/N = 347/355) in the intervention hospitals and 99.5% (n/N = 365/367) and 99.7% (n/N = 352/353) in the control hospitals respectively (**Figure 1**, Panel A).

The proportion of women with vaginal deliveries receiving antibiotics despite no indication (*e.g*. no prolonged rupture of membranes, fever, pneumonia, urinary tract infection) was 6.4% in control and intervention hospitals in 2019, and 7.8% (n/N = 18/230) in 2021, and by 2023 increasing till 10.6% (n/N = 23/216), respectively (**Figure 1**, Panel B).

Antibiotic prophylaxis for planned and emergency caesarean sections was 94.6% (n/N = 35/37) in the intervention hospitals and 100% (n/N = 47/47) in the control hospitals in 2019. In 2021 and 2023, in the intervention hospitals, it was up to 92.7% (n/N = 139/150) and 96.3% (n/N = 129/134), respectively, and in the control hospitals, it was up to 95.2% (n/N = 99/104) and 91.3% (n/N = 105/115) (**Figure 1**, Panel C).

In the intervention hospitals the proportion of planned caesarean section in women before the 39 + 0 weeks of gestation was 44.8% (n/N = 26/58) in 2021 and 28.3% (n/N = 17/60) in 2023; the proportion in the control hospitals was 50.0% (n/N = 8/16) in 2019, 53.3% (n/N = 16/30) in 2021, and 50.0% (n/N = 21/42) in 2023 (**Figure 1**, Panel D).

A summary of findings for maternal care is presented in Table S2 in the **Online Supplementary Document**.

### Newborn care

**Figure 2:**
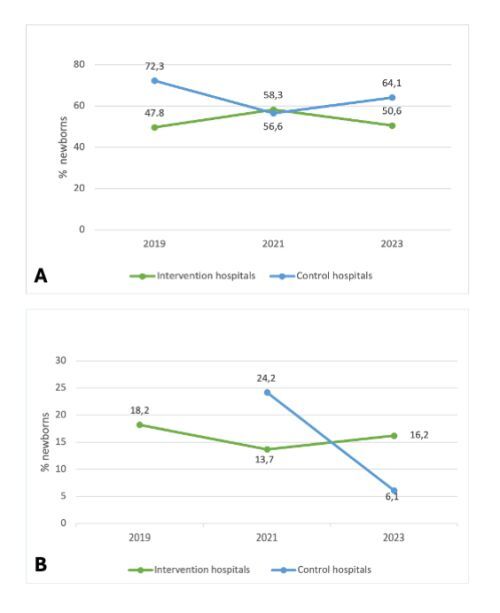
Quality indicators for newborn care. **Panel A.** Breastfeeding within 30 minutes of life. **Panel B.** Unjustified antibiotics.

The proportion of newborns breastfed within the first 30 minutes of life in the intervention hospitals was 47.8% (n/N = 214/448) in 2019, 58.3% (n/N = 261/448) in 2021, and 50.6% (n/N = 226/448) in 2023, and in the control hospitals 72.3% (n/N = 402/556), 56.6% (n/N = 314/556) and 64.1% (n/N = 356/556), respectively (**Figure 2**, Panel A).

The proportion of newborns who were unnecessarily prescribed antibiotics in the intervention hospitals was 18.2% (n/N = 4/22) in 2019, 13.7% (n/N = 7/51) in 2021, and 16.2% (n/N = 6/37) in 2023. In control hospitals the proportion of unjustified use of antibiotics was one out of three babies in 2019 (not shown in the graph due to small denominator), 24.2% (n/N = 8/33) in 2021, and 6.1% (n/N = 2/33) in 2023 (**Figure 2**, Panel B).

A summary of findings for newborn care is presented in Table S3 in the **Online Supplementary Document**.

### Child care

The characteristics of children included from the intervention and control hospitals are shown in **Table 1**. A summary of findings for child care is presented in Table S4 in the **Online Supplementary Document**.

**Table 1:** Basic characteristics of children*

	All hospitals	Intervention hospitals	Control hospitals
Age, in months			
*Median (IQR)*	14 (7–26)	13 (6–25)	14 (7–26)
*Infants (2–11)*	696 (43.7)	382 (45.6)	314 (41.5)
Referral from primary health care facilities or other hospitals	813 (51.0)	434 (51.9)	379 (50.1)
Overnight hospitalisation (18:00–8:00)	366 (23.0)	213 (25.4)	153 (20.2)
Hospitalisation in the intensive care unit	72 (4.5)	57 (6.8)	15 (2.0)
Primary diagnosis at admission			
*Acute respiratory infection*	179 (11.2)	94 (11.2)	85 (11.2)
*Pneumonia*	918 (57.6)	562 (67.1)	356 (47.1)
*Acute bronchitis/bronchiolitis*	241 (15.1)	131 (15.7)	110 (14.6)
*Acute gastroenteritis*	255 (16.0)	50 (6.0)	205 (27.1)
Other diagnoses during hospitalisation			
*Anaemia*	550 (34.5)	318 (38.0)	232 (30.7)
*Respiratory failure*	325 (20.4)	260 (31.1)	65 (8.6)
*Seizure*	99 (6.2)	72 (8.6)	31 (4.1)
*Acute pharyngitis*	40 (2.5)	30 (3.6)	10 (1.3)
*Urinary tract infection or pyelonephritis*	26 (1.6)	17 (2.0)	9 (1.2)
*Congenital heart disease*	25 (1.6)	17 (2.0)	8 (1.1)

IQR – interquartile range

*Values are presented as n (%) unless otherwise specified.

**Figure 3:**
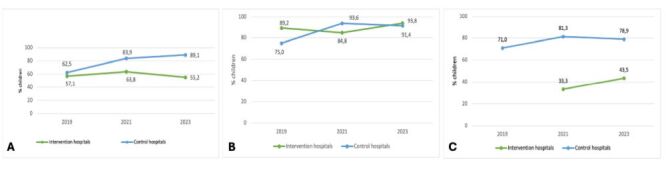
Quality indicators for child care. **Panel A.** Antibiotic prescription in children with an acute respiratory infection. **Panel B.** Antibiotic prescription in children with bronchitis/bronchiolitis. **Panel C.** Antibiotic prescription in children with acute gastroenteritis (excluding dysentery).

Antibiotic prescription for acute respiratory infection in the intervention hospitals was 57.1% (n/N = 4/7) in 2019, 63.8% (n/N = 37/58) in 2021, and 55.2% (n/N = 16/29) in 2023. For acute bronchitis or bronchiolitis, 89.2% (n/N = 33/37) of children received antibiotics in 2019, 84.8% (n/N = 39/46) in 2021, and 93.8% (n/N = 45/48) in 2023. In the control hospitals, antibiotic prescription for acute respiratory infection was 62,5% (n/N = 5/8) in 2019, 83.9% (n/N = 26/31) in 2021, and 89,1% (n/N = 41/46) in 2023, and 75.0% (n/N = 21/28), 93.6% (n/N = 44/47), and 91.4% (n/N = 32/35), retrospectively, in children with acute bronchitis or bronchiolitis (**Figure 3**, Panels A–B).

The proportion of children with diarrhoea (children with dysentery excluded) who were prescribed antibiotics in intervention hospitals was 33.3% (n/N = 8/24) in 2021 and 43.5% (n/N = 10/23) in 2023, and 71.0% (n/N = 22/31), 81.3% (n/N = 78/96), and 78.9% (n/N = 60/76) in control hospitals, retrospectively (**Figure 3**, Panel C).

In the intervention hospitals, blood oxygen saturation was measured using a pulse oximeter in 90.0% (n/N = 108/120) of children with a respiratory illness in 2019, 84.6% (n/N = 275/325) in 2021, and 96.2% (n/N = 329/342) in 2023. In control hospitals, oxygen saturation was measured in 69.7% (n/N = 62/89) of children presenting with a respiratory illness in 2019, 67.1% (n/N = 149/222) in 2021 and 58.3% (n/N = 140/240) in 2023 (Figure S2 in the **Online Supplementary Document**).

Children with pneumonia in intervention hospitals were prescribed corticosteroids in 35.5% (n/N = 27/76) of cases in 2019 and 54.7% (n/N = 145/265) in 2023. In control hospitals 28.3% (n/N = 15/53) of children with pneumonia received corticosteroids in 2019, and 50.9% (n/N = 81/159) in 2023 (Figure S3 in the **Online Supplementary Document**).

In both intervention and control hospitals, the proportion of children with diarrhoea who were prescribed ORS increased between the start and the end of the WHO quality improvement project, up to 66.7% (n/N = 16/24) and 71.1% (n/N = 54/76), respectively (Figure S4 in the **Online Supplementary Document**).

The proportion of children with diarrhoea receiving zinc in the intervention hospitals was 15.4% (n/N = 4/26) before the start of the QI project (2021) and 29.2% (n/N = 7/24) by the end of the QI project in 2023. In the control hospitals, not a single child with diarrhoea received zinc in 2023 (Figure S5 in the **Online Supplementary Document**).

## DISCUSSION

We performed an independent evaluation of the effect of a quality improvement project on maternal and newborn health in the Kyrgyz Republic. The project is described in detail elsewhere [14–17]. To evaluate the effect of the intervention independently, we recruited nine control hospitals matching the nine interventions hospitals, performed both a longitudinal analysis and comparison between the intervention and control hospitals. As there might have been an effect of COVID-19 in 2021, we also went back to 2019, to document the situation before the COVID-19 epidemic. For review, we selected reasonably common conditions, which should have been affected by the capacity building efforts during the intervention, to make sure we got comparable numbers. The effects of the interventions we documented in this way were overall not as large as we hoped for, and sometimes paradoxical between intervention and control hospitals.

We found a reduction of caesarean section rates, better use of pulse oximeters, and more use of zinc in children with diarrhoea. Antibiotic use somehow improved in unspecified acute respiratory infections, and was better in intervention hospitals for diarrhoea, but there was an imbalance at baseline already. Use of antibiotics remained high for neonates and children with bronchitis or bronchiolitis, where no antibiotics would have been needed, according to the guidelines [6]. Paradoxically, the situation got better in the control hospitals, as it did for breastfeeding. Some improvement was seen in the use of corticosteroids, but still half of the children with pneumonia received corticosteroids, a long-standing practice in the region [10].

Pulse oximetry was one of the foci of this and the previous projects [18], and also reinforced through the attention paid to oxygen saturation in the context of COVID-19. Comfortingly, this item improved in the intervention hospitals whereas it dropped in control hospitals after the 2021 assessment. The overuse of antibiotics in the country has been observed in other studies before and is common elsewhere too [5,19-21]. Giving unjustified antibiotic therapy to newborns can lead to irreversible consequences, intestinal dysbiosis, disruption of microflora colonisation, increased risk of atopy, candidiasis, ototoxic and nephrotoxic effects, and development of multi-resistance of the pathogens, but for health workers there is often the perception of it to be the safer option [22].

Caesarean section rates continue to increase worldwide. Caesarean section can save lives. However, caesarean sections are often performed without medical indications. The endline assessment results described elsewhere highlighted that the surgical technique for caesarean section achieved international standards [9]. It is comforting to note that the project had an impact on the more rational use of caesarean sections. Initiating breastfeeding within half an hour of delivery is one of the core interventions emphasised in the training, as delaying breastfeeding increases newborn mortality. It is therefore disappointing that rates remained at around 50%, and control hospitals, without the intervention, performed rather better. According to the National Clinical Protocol for Childbirth, oxytocin should be prescribed within the first minute after the birth of the child. This standard was met at fairly high levels in both intervention and control hospitals. Even though zinc is included in all clinical guidelines and protocols, the prescription of this drug remains at a low level. This may be due to the lack of the proper form of zinc in the country. It is worthwhile to note that the intervention hospitals made an effort to improve the prescription of zinc, over the control hospitals, where zinc was never given.

As mentioned, this study was embedded into a complex improvement project. As this project did not have a control arm in the approach, this study adds value by having the controls, with hospitals which were not formally part of the intervention. Similarly, the main before-and after comparisons in the intervention hospitals showed variable improvement across categories, but in general there was often an improvement from substandard categories to somewhat better performance [14–17]. To facilitate the use of these findings in practice, the results were presented to the Ministry of Health, hospital directors and frontline health workers, leading to the development of a concrete action plan within existing quality-of-care improvement cycles [8,11]. This process provides a practical pathway for clinicians to translate the evidence into routine practice.

Improving quality of care, addressing outdated behaviours, has been found to be a difficult task globally. Emphasis of development projects was often on the provision of training, but this has been shown to ineffective by itself [23,24]. We therefore embarked on a complex intervention including training, supportive supervisions, collaborative quality improvement meetings and strengthening the quality improvement committees [25]. The moderate success demonstrated here is in line with recent systematic reviews, which conclude that many strings need to be pulled, but still miracles cannot be expected [23,26,27].

A limitation of the study is that segmented regression was not used to adjust for potential seasonal patterns or secular trends. Because the analysis was designed primarily to explore overall trends rather than to estimate precise effect sizes, confidence intervals were not prespecified for reporting. The study relied on retrospective medical record review, therefore only what was recorded could be evaluated, and some important information might not have been documented in the patient files. Missing or undocumented data were not accounted for and may have introduced bias. Although these issues likely affected both intervention and control hospitals similarly, they may have reduced the observed effect size. The study also highlights the problems of an implementation research study in a small country with few hospitals. Matching of the controls was sometimes not ideal, with some observed difference in practice at baseline already. Where paradoxical effects happened, we can only speculate. One explanation might be contamination of the improvement approaches to control hospitals. A charismatic clinical leader in a control hospital who heard about better practices suggested for the country and implemented it in the hospital might explain this. A strength was that the study was conducted by independent experts with practical experience and knowledge of relevant guidelines and standards. Data were collected from 18 hospitals across the country, with different conditions, data collection was carried out randomly, following the data collection methodology, thus adding value to the before and after comparison of the main interventions trial.

## CONCLUSIONS

In conclusion, we could show independently that some improvement of clinical practices could be achieved in the context of the intervention study. Success was moderate overall, and pre-existing practice like the over-prescription of antibiotics and prescribing corticosteroids without a good reason could often not be addressed with a major improvement effect. The project established a quality improvement process, which will be a basis to build on further improvement interventions. Success requires persistent interventions, monitoring and the addition of further supportive measures.

## Additional material


Online Supplementary Document
