# Supplementary material for: Quality of medical services provided to mothers, newborns and children at the hospital level in the Kyrgyz Republic
Source: J Glob Health. 2026 Mar 20;16:04109. doi: 10.7189/jogh.16.04109 (PMC13003886; doi:10.7189/jogh.16.04109)
Supplement: Online Supplementary Document [file jogh-16-04109-s001.pdf]

Supplement to: Zholdosheva I, Nazhimidinova G, Akmatova B, Allakhveranova R, Tilenbaeva N, Weber MW, Jullien S. Quality of medical services provided to mothers, newborns and children at the hospital level in the Kyrgyz Republic. J Glob Health. 2026;16:04109.

Table S1. Primary outcomes and definitions

Area of care	Indicator
**MOTHERS**	
Post-partum uterotonics for post-partum haemorrhage prevention	Proportion of all women who gave birth in the health facility who received an uterotonic within 1 min of the birth of their baby (for prevention of post-partum haemorrhage)
Unjustified antibiotics	Proportion of women with vaginal delivery receiving antibiotics despite no indication (e.g., no prolonged rupture of membrane, no fever, no pneumonia, no urine tract infection)
Antibiotics and caesarean section	Proportion of women who received prophylactic antibiotics before caesarean section
Caesarean sections	Proportion of women undergoing planned caesarean section before 39+0 weeks' gestation
**NEWBORNS**	
Breastfeeding	Proportion of newborns who were breastfed during the first hour of life
Use of antibiotics	Proportion of newborns in the post-partum department (in rooming-in with mother) receiving antibiotics
**CHILDREN**	
Antibiotic prescription for acute respiratory infection	Proportion of children 2–59 months of age with pneumonia or severe pneumonia who received correct antibiotic treatment (antibiotic, dose, frequency and duration) according to WHO guidelines[1]
Antibiotic prescription for diarrhoea	Proportion of children 2–59 months of age hospitalized with a primary diagnosis of diarrhoea (acute gastroenteritis), dysentery excluded, who were prescribed antibiotics
Use of pulse oximeter	Proportion of children 2–59 months of age with a primary diagnosis of acute respiratory infection with oxygen saturation recorded upon admission
Corticosteroids prescription for pneumonia	Proportion of children 2–59 months of age hospitalized with a primary diagnosis of pneumonia who were prescribed corticosteroids
ORS and zinc in diarrhoea	Proportion of children managed for diarrhoea who were prescribed ORS, zinc supplementation, or both

[1]World Health Organization. Pocket book of Hospital care for children. Guidelines for the management of common childhood illnesses: Second edition. Geneva, Switzerland: World Health Organization; 2013. Available: https://www.who.int/publications/i/item/978-92-4-154837-3. Accessed: 23 January 2026.

Table S2. Summary of findings for maternal care

Indicators	Year	Intervention hospitals	Control hospitals
Uterotonics within one minute of delivery	2019	95,7% (111/116)	100% (160/160)
	2021	97,8% (349/353)	99,5% (365/367)
	2023	97,9% (347/355)	99,7% (352/353)
Women with vaginal deliveries receiving antibiotics despite no indication (e.g. no prolonged rupture of membranes, fever, pneumonia, urinary tract infection)	2019	6,4% (5/78)	6,4% (7/110)
	2021	6,5% (13/201)	7,8% (18/230)
	2023	10,6% (23/216)	7,8% (18/230)
Antibiotic prophylaxis for planned and emergency caesarean sections	2019	94.6% (35/37)	100% (47/47)
	2021	92.7% (139/150)	95.2% (99/104)
	2023	96.3% (129/134)	91.3% (105/115)
Planned caesarean section in women before the 39+0 weeks of gestation	2019	40,0% (8/20)	50,0% (8/16)
	2021	44,8% (26/58)	53,3% (16/30)
	2023	28,3% (17/60)	50,0% (21/42)

Table S3. Summary of findings for newborn care

Indicators	Year	Intervention hospitals	Control hospitals
Breastfeeding within the first 30 minutes of life	2019	47.8% (214/448)	72.3% (402/556)
	2021	58.3% (261/448)	56.6% (314/556)
	2023	50.6% (226/448)	64.1% (356/556)
Unjustified prescription of antibiotics	2019	18.2% (4/22)	(1/3)[a]
	2021	13.7% (7/51)	24.2% (8/33)
	2023	16.2% (6/37)	6.1% (2/33)

[a] Proportion not shown due to small denominator

Table S4. Summary of findings for child care

Indicators	Year	Intervention hospitals	Control hospitals
Antibiotic prescription in children with a respiratory infection	2019	57.1% (4/7)	62,5% (5/8)
	2021	63.8% (37/58)	83.9% (26/31)
	2023	55.2% (16/29)	89,1% (41/46)
Antibiotic prescription in children with bronchitis/ bronchiolitis	2019	89.2% (33/37)	75.0% (21/28)
	2021	84.8% (39/46)	93.6% (44/47)
	2023	93.8% (45/48)	91.4% (32/35)
Antibiotic prescription in children with acute gastroenteritis (excluded dysentery)	2019	-	71.0% (22/31)
	2021	33.3% (8/24)	81.3% (78/96)
	2023	43.5% (10/23)	78.9% (60/76)
Use of pulse oximetry in children with respiratory infections	2019	90.0% (108/120)	69.7% (62/89)
	2021	84.6% (275/325)	67.1% (149/222)
	2023	96.2% (329/342)	58.3% (140/240)
Corticosteroids prescription in children with pneumonia	2019	35.5% (27/76)	28.3% (15/53)
	2021	67.9% (150/221)	52.8% (76/144)
	2023	54.7% (145/265)	50.9% (81/159)
Oral rehydrated salts (ORS) prescription in children with diarrhoea	2019	-	35.5% (11/31)
	2021	53.8% (14/26)	61.2% (60/98)
	2023	66.7% (16/24)	71.1% (54/76)
Zinc prescription in children with diarrhoea	2019	-	32,3% (10/31)
	2021	15.4% (4/26)	3.1% (3/98)
	2023	29.2% (7/24)	0.0% (0/76)

Figure S1. Selection of hospitals and medical records

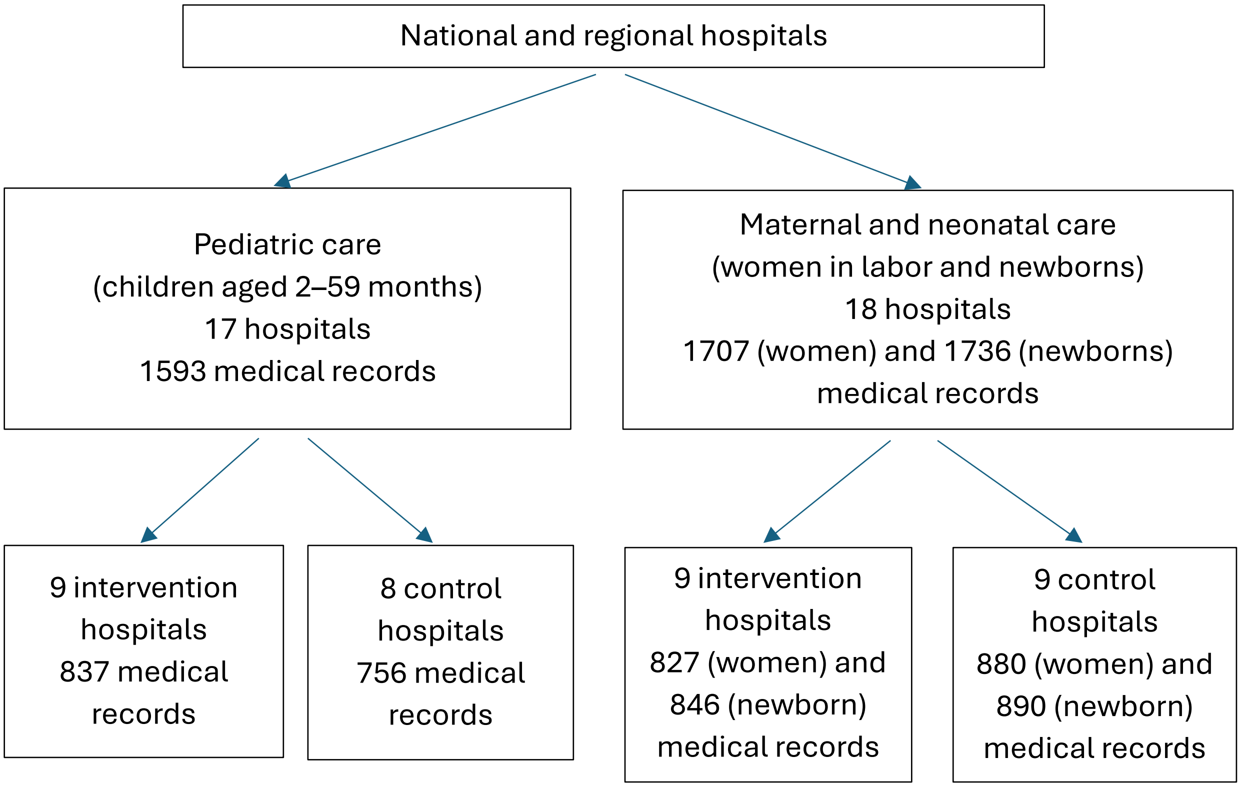

Figure S2. Pulse oximetry use in children with a respiratory infection.

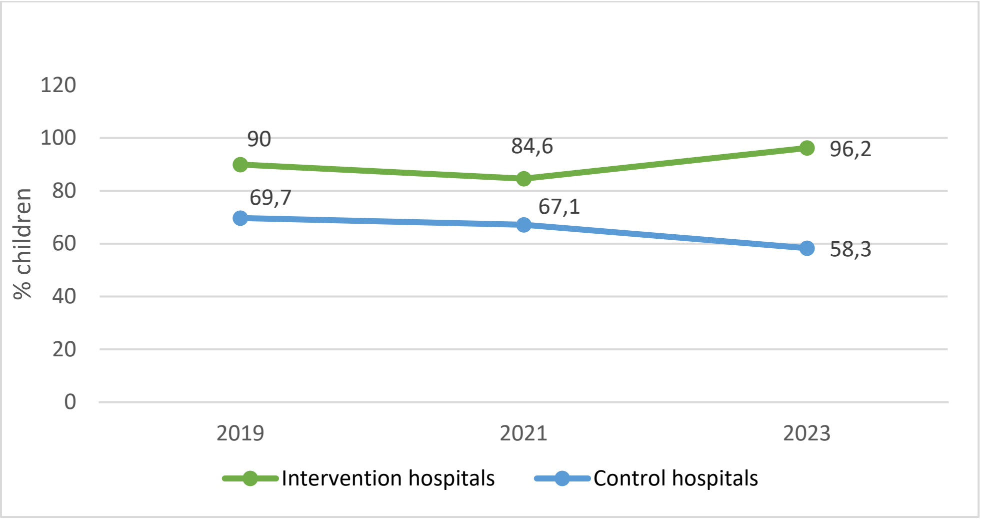

Figure S3. Corticosteroids prescription in children with pneumonia.

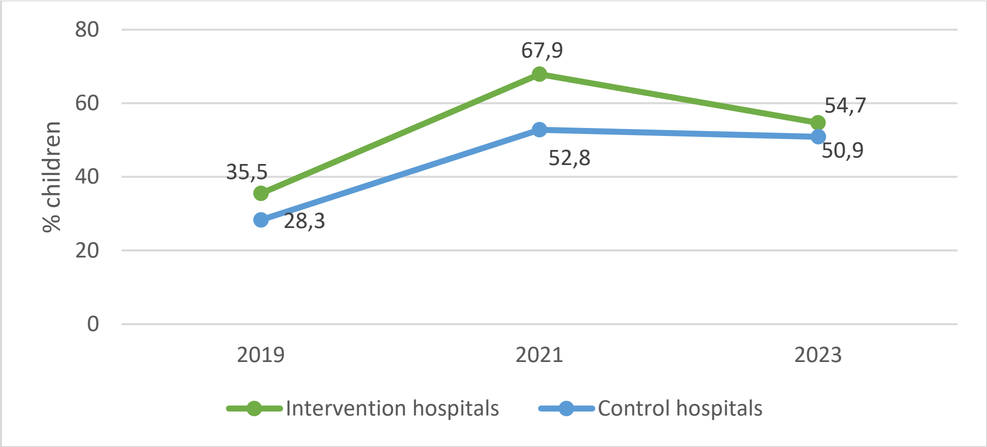

Figure S4. Oral rehydration salts prescription in children with diarrhoea.

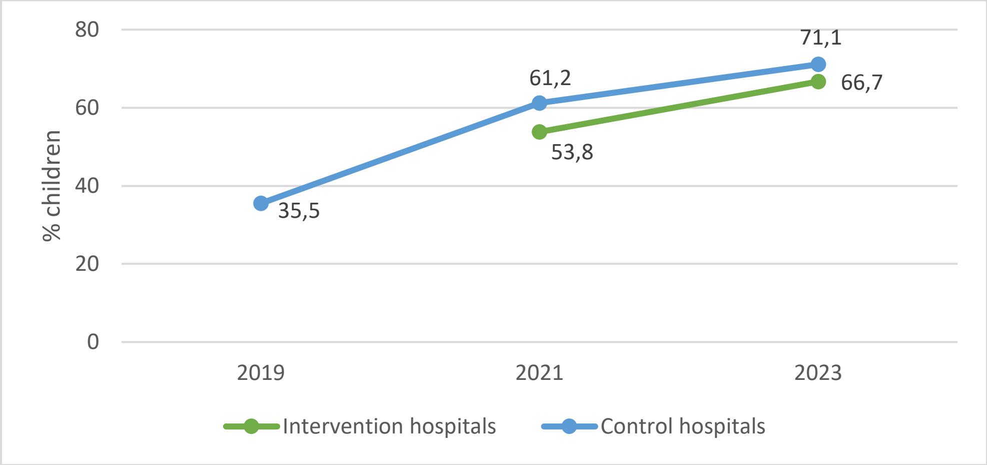

Figure S5. Zinc prescription in children with diarrhoea.

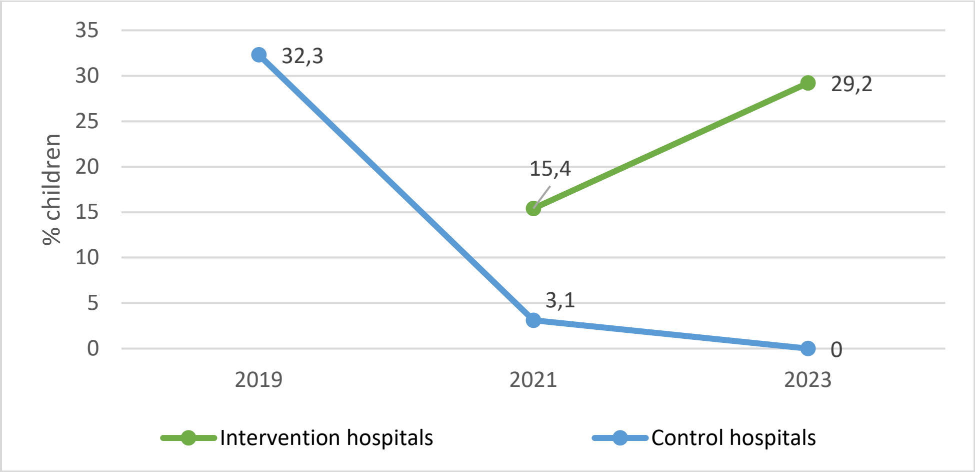

Text S1. Explanation of authorship change statement

The original authorship contributions statement supplied in the submitted version of this article disclosed that SJ conceived the assessment, designed the study and developed the project proposal. She was also involved in the implementation of the study. However in the initial submission, SJ was inadvertently not recognized as a co-corresponding author with RA in the authorship byline, while RA was also not recognized in the implementation of the study. The authors noted this issue at the review stage and notified the Journal of Global Health's editorial team. All authors consented to this change by signing an agreement form.
